# Asylum Seekers’ Responses to Government COVID-19 Recommendations: A Cross-sectional Survey in a Swiss Canton

**DOI:** 10.1007/s10903-022-01436-3

**Published:** 2022-12-12

**Authors:** Kevin Morisod, Marie-Anne Durand, Kevin Selby, Marie-Annick Le Pogam, Véronique S. Grazioli, Javier Sanchis Zozaya, Patrick Bodenmann, Christian von Plessen

**Affiliations:** 1 Department of Vulnerabilities and Social Medicine, Center for Primary Care and Public Health (Unisanté), Lausanne, Switzerland; 2 Chair of Medicine for Vulnerable Populations, University of Lausanne, Lausanne, Switzerland; 3 CERPOP, Université de Toulouse, Inserm, UPS, Toulouse, France; 4 Center for Primary Care and Public Health (Unisanté), Lausanne, Switzerland; 5 Department Training, Research and Innovation, Center for Primary Care and Public Health (Unisanté), Lausanne, Switzerland; 6 Department of Epidemiology and Health Systems, Center for Primary Care and Public Health (Unisanté), Lausanne, Switzerland; 7 Department of Ambulatory Care, Center for Primary Care and Public Health (Unisanté), Lausanne, Switzerland; 8 Direction Générale de La Santé (DGS), Lausanne, Switzerland; 9 Department of Clinical Research, University of Southern Denmark, Odense, Denmark; 10 Department of Psychiatry, Lausanne University Hospital (CHUV), Lausanne, Switzerland

**Keywords:** Asylum seekers, COVID-19, Public health recommendations, Health equity

## Abstract

Asylum seekers face multiple language, cultural and administrative barriers that could result in the inappropriate implementation of COVID-19 measures. This study aimed to explore their knowledge and attitudes to recommendations about COVID-19. We conducted a cross-sectional survey among asylum seekers living in the canton of Vaud, Switzerland. We used logistic regressions to analyze associations between knowledge about health recommendations, the experience of the pandemic and belief to rumors, and participant sociodemographic characteristics. In total, 242 people participated in the survey, with 63% of men (n = 150) and a median age of 30 years old (IQR 23–40). Low knowledge was associated with linguistic barriers (aOR 0.36, 95% CI 0.14–0.94, p = 0.028) and living in a community center (aOR 0.43, 95% CI 0.22–0.85, p = 0.014). Rejected asylum seekers were more likely to believe COVID-19 rumors (aOR 2.81, 95% CI 1.24–6.36, p = 0.013). This survey underlines the importance of tailoring health recommendations and interventions to reach asylum seekers, particularly those living in community centers or facing language barriers.

**Supplementary Information:**

The online version contains supplementary material available at 10.1007/s10903-022-01436-3.

## Background

The burden of the COVID-19 pandemic is notably high among migrant populations—especially asylum seekers and refugees worldwide [1–5]. Preliminary data highlighted the need to consider social context and living conditions, as high population density, belonging to a minority ethnic group, or social deprivation are risk factors for contracting SARS-CoV-2 infection [6–11]. A recent systematic review by Hayward et al. found, for example, that asylum seekers and refugees are at increased risk of infection and have been disproportionately affected by the COVID-19 pandemic. [12]

Among the different factors associated with the additional burden of the pandemic on asylum seekers, poor access to COVID-19 health recommendations seems to play an important role. A recent report by the International Organization for Migration (IOM) confirmed that limited awareness of public health recommendations due to linguistic and cultural barriers was a vulnerability factor for asylum seekers [13–15]. Similarly, lower access to healthcare systems, including mental health, and the fear of legal repercussions increased the risk of health inequities [12, 14–16]. For example, asylum seekers living in community centers during the pandemic face somatic and mental health challenges which require specific public health recommendations [15]. Reception centers are indeed characterized by crowded living conditions, shared rooms and little or no privacy, which could increase both the risks and the fears of being infected [17–20]. Accordingly, a recent systematic review advocates for better consideration of asylum seekers living in reception centers during the pandemic [8] and urge the need for adapted public health measures [12, 21–24] .

The current literature suggests that linguistic and cultural barriers, poor health literacy, living conditions, and legal status could contribute to mistrust of authorities and increase the COVID-19 pandemic burden among asylum seekers and refugees [25–27]. However, there is little current data on asylum seekers' access to and understanding of health recommendations and their perception of the COVID-19 pandemic. Moreover, the experience of the pandemic and the understanding of health measures among asylum seekers might vary according to place of living (community center vs private apartment), legal status, level of proficiency in French (the official language of the Swiss Canton studied) or health literacy. We, therefore, aimed to explore asylum seekers’ attitudes and knowledge concerning COVID-19 recommendations and to describe associations between these variables and participants’ socio-demographic characteristics.

## Methods

### Participants and Data Collection

We conducted a self-administrated cross-sectional survey about participants’ knowledge, attitudes and perceived adherence to recommendations about COVID-19. Most survey questions were adapted from an online survey of the general population of the Canton of Vaud [28]. We simplified the language of the questions to a lower readability level in English. Then we translated it into the nine most common languages among asylum seekers residing in the Canton of Vaud: French, Tigrinya, Dari, Arab, Somali, Georgian, Tamil, Albanian and Serbo-Croatian. We translated the English questionnaire into these nine languages with the help of bilingual medical and nursing students from a local NGO and community interpreters. Except for Tigrinya and Tamil, a second translator proofread each translation.

We included asylum seekers, defined as asylum applicants with a pending procedure (N permit in Switzerland), as temporarily admitted (F permit), fully admitted (B permit) or rejected (emergency aid) residing in the Canton of Vaud. We excluded children under 18 years old, individuals not living in the Canton of Vaud, and former asylum seekers with a settlement permit (C Permit). Asylum seekers who cannot read or write were also excluded. In October 2020, according to the cantonal administrative data, 744 asylum seekers lived in one of the ten cantonal asylum community centers.

We identified 29 NGOs helping asylum seekers in the Canton of Vaud. We contacted them by email and phone to present the study and the survey questionnaires. We also worked closely with the persons in charge of the community centers in the canton of Vaud. We organized visits to all the centers to present the study and questionnaires to the residents. Finally, the questionnaire was also available online with a link sent to all study partners, including the identified NGOs.

The first page of the survey provided information in the selected language explaining that the study would like to know how they feel about the COVID-19 public health recommendations to improve the canton response and help research in this area. We also informed participants that the survey was anonymous and voluntary, and that they would not be contacted again. No incentive was used to encourage participation. The questionnaire took 15–20 min to complete.

We distributed the questionnaires (online and paper form) and collected data between August and October 2020. Online questionnaires were developed using the REDCap web application. We added the paper form data to the REDCap database in a second step. At the time of the data collection, the following health measures were in force in Switzerland: wearing masks in public transport, respecting social distance of 1.5 m, encouragement of hand hygiene, and recommended home office work. In addition, quarantine and isolation measures were mandatory.

All procedures were conducted following the ethical standards of the Human Research Ethics Committee of Canton de Vaud and the Swiss Law on Human Research. As all data collected were anonymous, an ethics approval by the Ethics Committee was not required. (Article 2 of the Swiss Law on Human Research).

### Measures

#### Sociodemographic Characteristics

Sociodemographic characteristics collected included age, gender, level of education, French language proficiency, adapted and translated versions of a validated health literacy item [29], place of living (community centers vs private apartments) and legal status. The legal status variable is a dichotomization of the participant into two groups: the one with a permit (N permit, F permit or B permit) and the one with the *Emergency aid* status (rejected asylum seekers). This group represents indeed a particularly vulnerable category of asylum seekers and refugees as their legal status in Switzerland is highly insecure.

#### COVID-19 Data

The following questions asked participants whether they had been tested positive for COVID-19, were part of a group at risk (defined as people with comorbidities such as hypertension, diabetes, heart or lung problems or weaker immune system) and knew what to do if they had COVID-19 symptoms.

**Fig. 1:**
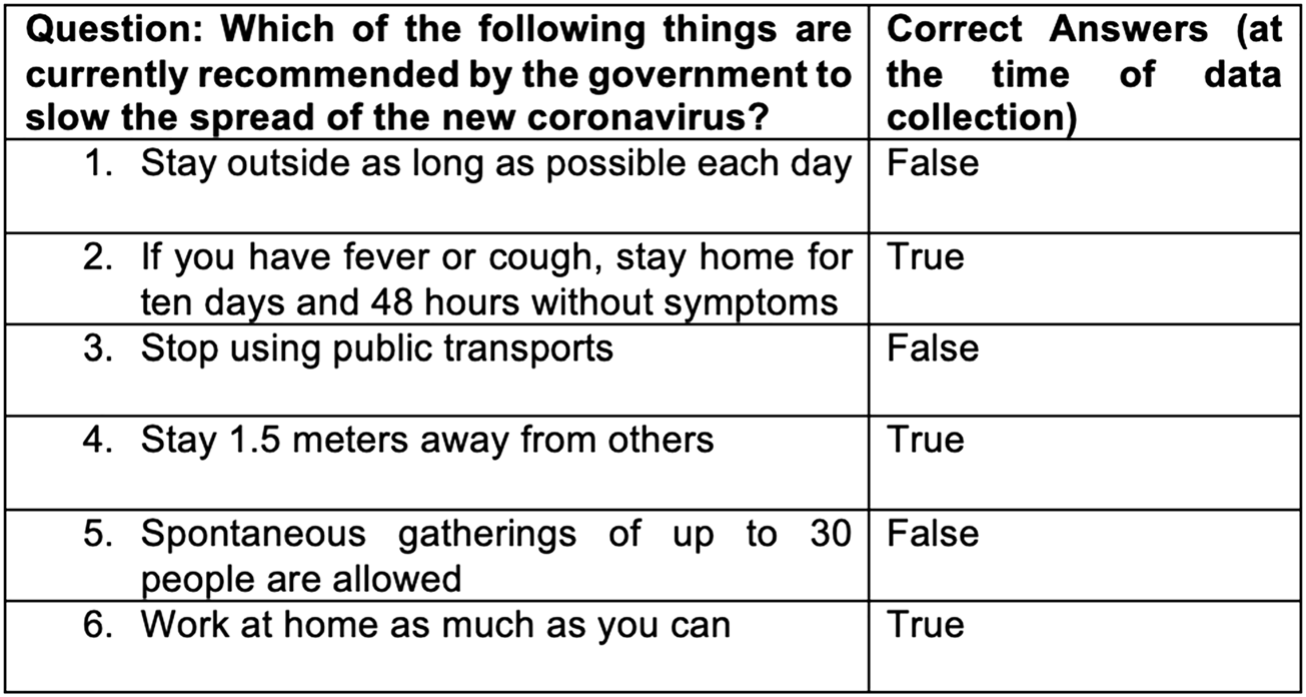
Knowledge score on six true/false questions

**Fig. 2:**
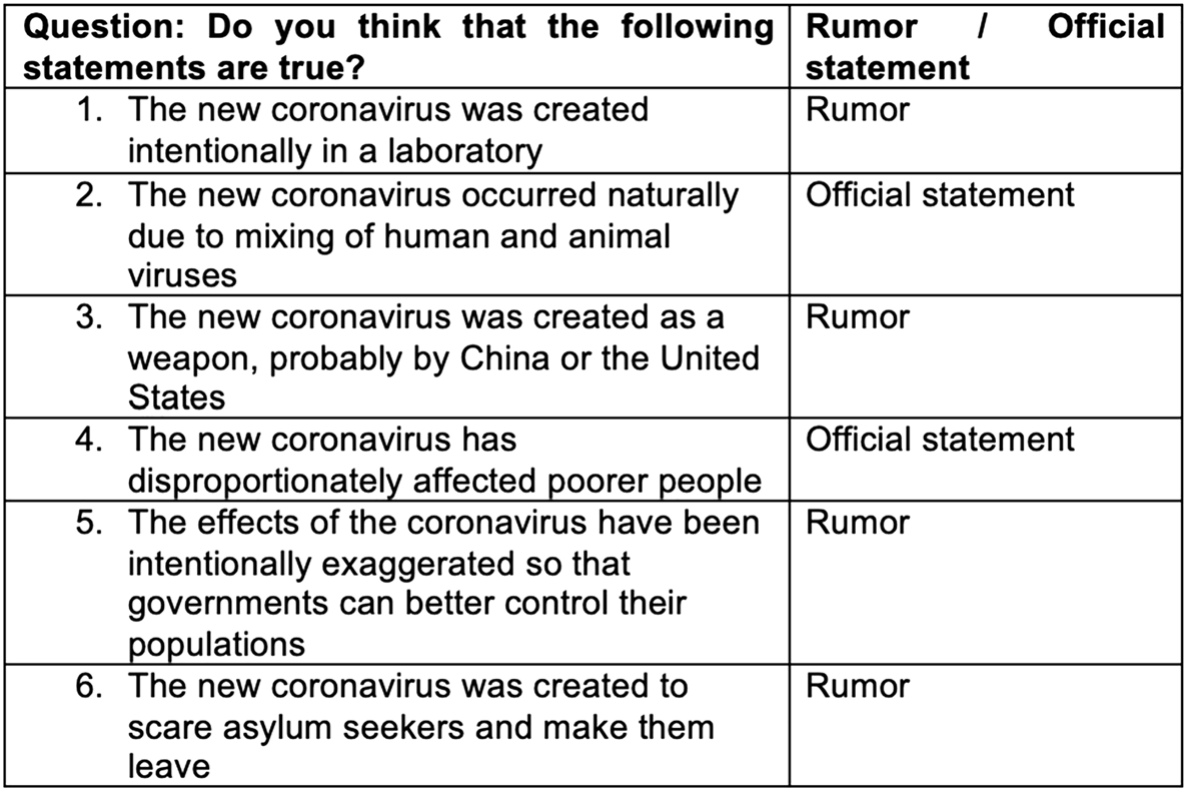
COVID-19 rumors and official statement

Then, the questions investigated the participants' understanding of the COVID-19 pandemic and public health recommendations. A knowledge score was developed with six true–false items about current government recommendations adapted from a previously published survey [28] (Fig. 1). Participants were also invited to answer six statements regarding COVID-19 rumors (Fig. 2). Visual analogue scores were used to measure self-reported adherence and perception of government measures.

The survey finally assessed the means of access to COVID-19 information and why participants stopped following the recommendations (Supplementary Information).

### Statistical Analysis

We described study participant characteristics and answers to the questions using frequency (n) and relative frequency (percentage) for categorical variables and median and interquartile range (IQR) for non-normally distributed continuous variables (mean and standard deviation otherwise).

We used logistic regressions to explore associations between the outcomes of interest and participant characteristics, such as place of living (community center or private apartment), legal status (asylum seekers with a permit vs rejected asylum seekers), health literacy (high vs low health literacy), education level (high vs low education level) or official language proficiency (high vs low French proficiency). Regression models were adjusted for age, gender and relevant confounders. Models’ calibration was tested using the Hosmer–Lemeshow goodness-of-fit test. Associations with a p-value < 0.05 were considered statistically significant. Missing values were assumed to be missing at random. All analyzes were performed with STATA version 16.

## Results

In total, 242 persons participated in the study. About two-thirds were men (n = 150), with a median age of 30 years old (IQR 23–40). Half of the participants (55%, n = 132) lived in a community center and 45% (n = 110) in a private apartment. The legal status of the participants was divided between participants with a permit (74%, n = 173) and participants with the *Emergency aid* status (26%, n = 60). All languages of the questionnaire were used. In descending order, the languages used were French (34%, n = 82), Dari (18%, n = 44), Tigrinya (12%, n = 29), Arab (12%, n = 29), Tamil (6%, n = 15), English (6%, n = 14), Georgian (5%, n = 12), Somalian (3%, n = 8), Albanian (3%, n = 7) and Serbo-Croatian (0.5%, n = 1). Health literacy was low in 42% of the participants (n = 100), and 62% (n = 119) had a low to moderate level of education (compulsory education or apprenticeship). In addition, 35% (n = 83) of participants described a low level of French comprehension (see Table 1).

**Table 1:** Socio-demographic and COVID-19 related characteristics of participants (N = 242)

Characteristics	Value, n (%)
Age (years)	
18–39	176 (73)
40–64	54 (22)
≥ 65	12 (5)
Gender (2 missing)	
Female	90 (38)
Male	150 (62)
Legal status (9 missing)	
Asylum seekers with permit	173 (74)
Rejected asylum seekers	60 (26)
Education level (7 missing)	
Compulsory	59 (25)
Apprenticeship	60 (26)
High School	43 (18)
University	47 (20)
Don’t know	26 (11)
Health literacy^a^ (5 missing)	
High	137 (58)
Low	100 (42)
Place of living (0 missing)	
Community centre	132 (55)
Private apartment	110 (45)
French language proficiency (3 missing)^b^	
High	156 (65)
Low	83 (35)
Tested for Covid-19 (3 missing)	
Positive	11 (5)
Negative	25 (10)
Awaiting result	3 (1)
No	192 (80)
Don’t know	8 (3)
Social worker or community help (6 missing)	
Yes	99 (42)
No	128 (54)
Don’t know	9 (4)
At-risk (at least one comorbidity) (3 missing)	
Yes	40 (17)
No	172 (72)
Don’t know	27 (11)

^a^Dichotomized, “Often” and “Always” as high and “Never”, “Rarely”, “Sometimes” and “I don’t know” as low health literacy

^b^Dichotomized, “Very well” and “Well” as high, and “Not well”, “Not at all” and “I don’t know” as low French language proficiency

### Knowledge About COVID-19 Recommendations

In our study, only 43% (n = 104) of the participants had a high knowledge score (correctly answered at least 5 of the six questions of the knowledge score in Fig. 1), with a median score of 4/6. (See Table 2) After adjustment for age, gender and education, a lower knowledge score was associated with lower French language proficiency (aOR 0.34, 95% CI 0.13–0.89, p = 0.028) and living in a community center (aOR 0.41, 95% CI 0.20–0.84, p = 0.014), but not with health literacy level (aOR 1.52, 95% CI 0.81–2.84, p = 0.188).

**Table 2:** Non adjusted Odd Ratio of socio-demographic characteristics and knowledge, confidence and COVID-19 rumors (with 95% CI and p-value)

	Knowledge^a^	Confidence^b^	Rumors^c^ (overall)	Natural origin of COVID-19^d^	Control of population^e^
Gender (Female)	0.86 (0.51–1.47, p = 0.59)	0.86 (0.45–1.64, p = 0.65)	0.91 (0.53–1.56, p = 0.73)	0.75 (0.41–1.37, p = 0.35)	0.86 (0.39–1.87, p = 0.70)
Legal status (Rejected asylum seekers)	0.55 (0.30–1.03, p = 0.60)	0.80 (0.38–1.67, p = 0.55)	1.36 (0.75–2.46, p = 0.31)	0.38 (0.17–0.82, **p = 0.01**)	2.79 (1.28–6.09, **p = 0.01**)
Education level (Low education level)	1.13 (0.67–1.93, p = 0.64)	1.17 (0.60–2.89, p = 0.65)	1.59 (0.93–2.72, p = 0.09)	1.13 (0.63–2.01, p = 0.69)	1.12 (0.52–2.39, p = 0.77)
Health literacy (Low health literacy)	0.87 (0.51–1.46, p = 0.59)	0.44 (0.23–0.83, **p = 0.01**)	1.14 (0.67–1.92, p = 0.63)	0.58 (0.32–1.04, p = 0.07)	1.08 (0.51–2.28, p = 0.85)
Place of living (Community centers)	0.45 (0.27–0.75, **p < 0.01**)	0.30 (0.15–0.60, **p < 0.01**)	1.13 (0.67–1.90, p = 0.65)	0.49 (0.28–0.87, **p = 0.01**)	1.25 (0.59–2.67, p = 0.56)
French language proficiency (Low level)	0.43 (0.25–0.76, **p < 0.01**)	0.78 (0.40–1.50, p = 0.45)	0.81 (0.47–1.41, p = 0.46)	0.31 (0.16–0.62, **p < 0.01**)	0.70 (0.31–1.60, p = 0.40)
Social worker (absence of)	1.09 (0.65–1.84, p = 0.75)	1.09 (0.57–2.09, p = 0.80)	1.81 (1.05–3.12, **p = 0.03**)	1.33 (0.74–2.40, p = 0.34)	1.45 (0.66–3.16, p = 0.35)
Tested positive for COVID-19	0.49 (0.13–1.89, p = 0.30)	1.28 (0.27–6.12, p = 0.76)	2.89 (0.82–10.16, p = 0.09)	NA	1.47 (0.30–7.12, p = 0.64)
At-risk (at least one comorbidity)	1.03 (0.52–2.06, p = 0.93)	0.34 (0.16–0.73, **p < 0.01**)	1.07 (0.53–2.16, p = 0.85)	0.40 (0.16–1.00, p = 0.05)	0.65 (0.21–2.00, p = 0.46)

A p-value < 0.05 is considered statistically significant (in bold in the table)

^a^Comparison based on the knowledge score (Illustration 1) dichotomized into high knowledge (at least 5/6 correct answers) and low knowledge (< 5/6 correct answers)

^b^Comparison between participants about “Knowing what to do if sick or if COVID-19 symptoms”

^c^Comparison based on the answers of rumors questions (Illustration 2). Positive if adhesion to at least one COVID-19 rumor

^d^Comparison based on the official statement “The new coronavirus occurred naturally due to mixing of human and animal viruses”

^e^Comparison based on the COVID-19 rumor “The effects of the coronavirus have been intentionally exaggerated so that governments can better control their populations”

Similarly, participants living in a community center were less confident about what to do if they got COVID-19 symptoms (naOR 0.30, 95% CI 0.15–0.60, p < 0.01), as well as participants with low health literacy (naOR 0.44, 95% CI 0.23–0.83, p = 0.01). (See Table 2) After adjustment, confidence remained associated with place of living and health literacy.

### Access to Information About COVID-19 Recommendations

**Fig. 3:**
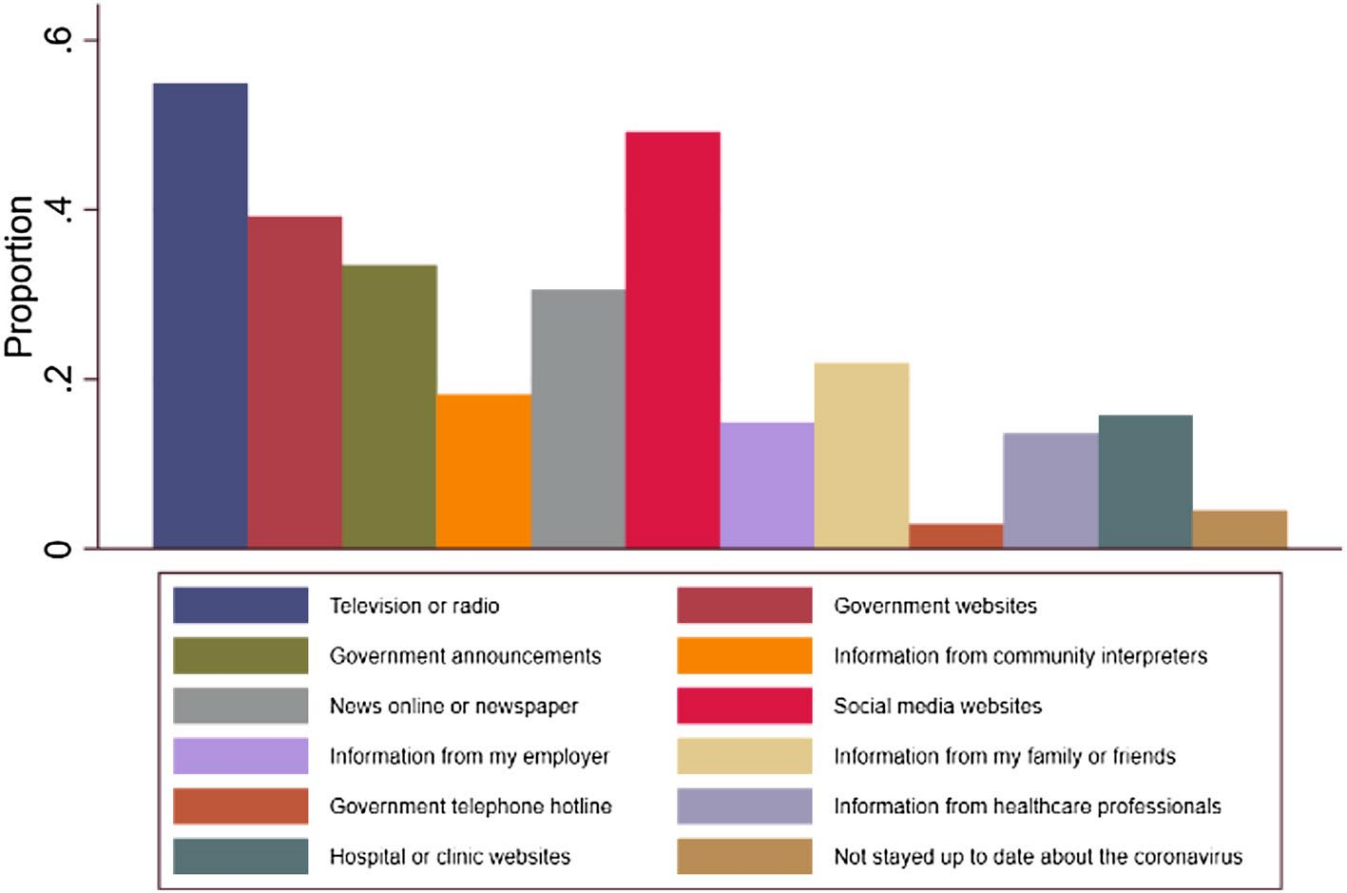
Means of access to COVID-19 recommendations

Most participants accessed information about COVID-19 recommendations on television (55%, n = 133), social media (49%, n = 119) and government websites (39%, n = 95) (Fig. 3).

Participants living in community centers were statistically less likely to use television as a means of information. However, they were twice as likely to have accessed information via community interpreters than participants living in private apartments (22.3% vs 9.6%). These differences were statistically significant after adjusting for age, gender and education level for the use of television (aOR 0.42, 95% CI 0.23–0.75, p = 0.003) and community interpreters (aOR 2.99, 95% CI 1.29–6.91, p = 0.011).

### Adherence to and Attitudes About COVID-19 Recommendations

Self-reported adherence to COVID-19 recommendations was high, with 67% of participants reporting a high degree (score > 80) and a median adherence of 95 (IQR 70.5–100). Adherence was lower for participants on emergency aid, although the difference wasn’t statistically significant (naOR 0.58, 95% CI 0.31–1.11, p = 0.10) (See Table 3).

**Table 3:** Non adjusted Odd Ratio of socio-demographic characteristics and opinion on government recommendations, adherence to recommendations and main reasons to stop following COVID-19 measures (with 95% CI and p-value)

	Measures too restrictive^a^	Measures not strong enough^b^	Adherence^c^	Main reason to stop 1^d^	Main reason to stop 2^e^	Main reason to stop 3^f^
Gender (Female)	0.69 (0.38–1.23, p = 0.21)	0.82 (0.27–2.49, p = 0.73)	0.96 (0.53–1.73, p = 0.89)	1.16 (0.59–2.30, p = 0.66)	1.23 (0.52–2.95, p = 0.64)	0.90 (0.50–1.60, p = 0.71)
Legal status (Rejected asylum seekers)	1.10 (0.59–2.06, p = 0.76)	1.88 (0.59–5.97, p = 0.29)	0.58 (0.31–1.11, p = 0.10)	1.07 (0.50–2.30, p = 0.86)	0.34 (0.15–0.76, **p < 0.01**)	1.60 (0.86–2.98, p = 0.14)
Education level (Low education level)	1.00 (0.57–1.77, p = 0.99)	3.50 (1.16–10.60, **p = 0.03**)	1.44 (0.80–2.61, p = 0.23)	1.24 (0.62–2.46, p = 0.55)	1.49 (0.83–2.68, p = 0.18)	0.94 (0.53–1.69, p = 0.85)
Health literacy (Low health literacy)	1.15 (0.66–2.01, p = 0.61)	2.16 (0.74–6.28, p = 0.16)	0.96 (0.54–1.71, p = 0.89)	1.31 (0.67–2.55, p = 0.43)	0.36 (0.19–0.69, **p < 0.01**)	0.73 (0.41–1.30, p = 0.28)
Place of living (Community centers)	1.91 (1.09–3.35, **p = 0.03**)	1.72 (0.57–5.20, p = 0.34)	0.82 (0.47–1.45, p = 0.50)	1.63 (0.82–3.25, p = 0.17)	0.46 (0.26–0.83, **p = 0.01**)	1.37 (0.78–2.40, p = 0.28)
French language proficiency (Low level)	1.28 (0.73–2.27, p = 0.39)	1.44 (0.48–4.30, p = 0.51)	1.28 (0.70–2.36, p = 0.42)	1.05 (0.53–2.11, p = 0.88)	0.48 (0.25–0.93, **p = 0.03**)	0.84 (0.46–1.52, p = 0.56)
Social worker (absence of)	0.94 (0.53–1.64, p = 0.82)	2.07 (0.64–6.71, p = 0.22)	0.99 (0.55–1.77, p = 0.97)	1.16 (0.58–2.31, p = 0.68)	2.13 (1.14–3.97, **p = 0.02**)	1.01 (0.57–1.79, p = 0.98)
Tested positive for COVID-19	0.48 (0.10–2.29, p = 0.36)	3.68 (0.72–18.78, p = 0.12)	0.59 (0.15–2.28, p = 0.45)	1.88 (0.48–7.39, p = 0.37)	0.61 (0.13–2.90, p = 0.53)	2.18 (0.64–7.41, p = 0.21)
At-risk (at least one comorbidity)	1.11 (0.53–2.32, p = 0.78)	1.47 (0.38–5.69, p = 0.58)	1.77 (0.78–4.01, p = 0.17)	1.23 (0.52–2.95, p = 0.64)	0.80 (0.35–1.80, p = 0.58)	0.21 (0.07–0.61, **p < 0.01**)

A p-value < 0.05 is considered statistically significant (in bold in the table)

^a^Satisfaction with government recommendations

^b^Ibidem

^c^A score > 80 for self-reported adherence were considered as high adherence

^d^Main reason 1 to stop following the COVID-19 measures: “My home is too small to stay inside all the time”

^e^Main reason 2 to stop following the COVID-19 measures: “I have to leave the house for food and essentials”

^f^Main reason 3 to stop following the COVID-19 measures: “I don’t have the choice (ex: must keep working or don’t have the means)

About 51% of participants found that the COVID-19 measures were “about right”, 11% found them not restrictive enough and 38% too restrictive. In a non-adjusted analysis, asylum seekers living in community centers considered the government COVID-19 measures as too restrictive (naOR 1.91, 95% CI 1.09–3.35, p = 0.03), whereas asylum seekers with low education level statistically significantly considered the measures as not strong enough (naOR 3.50, 95% CI 1.16–10.60, p = 0.03) (See Table 3).

### Reasons to Stop Following COVID-19 Recommendations

**Fig. 4:**
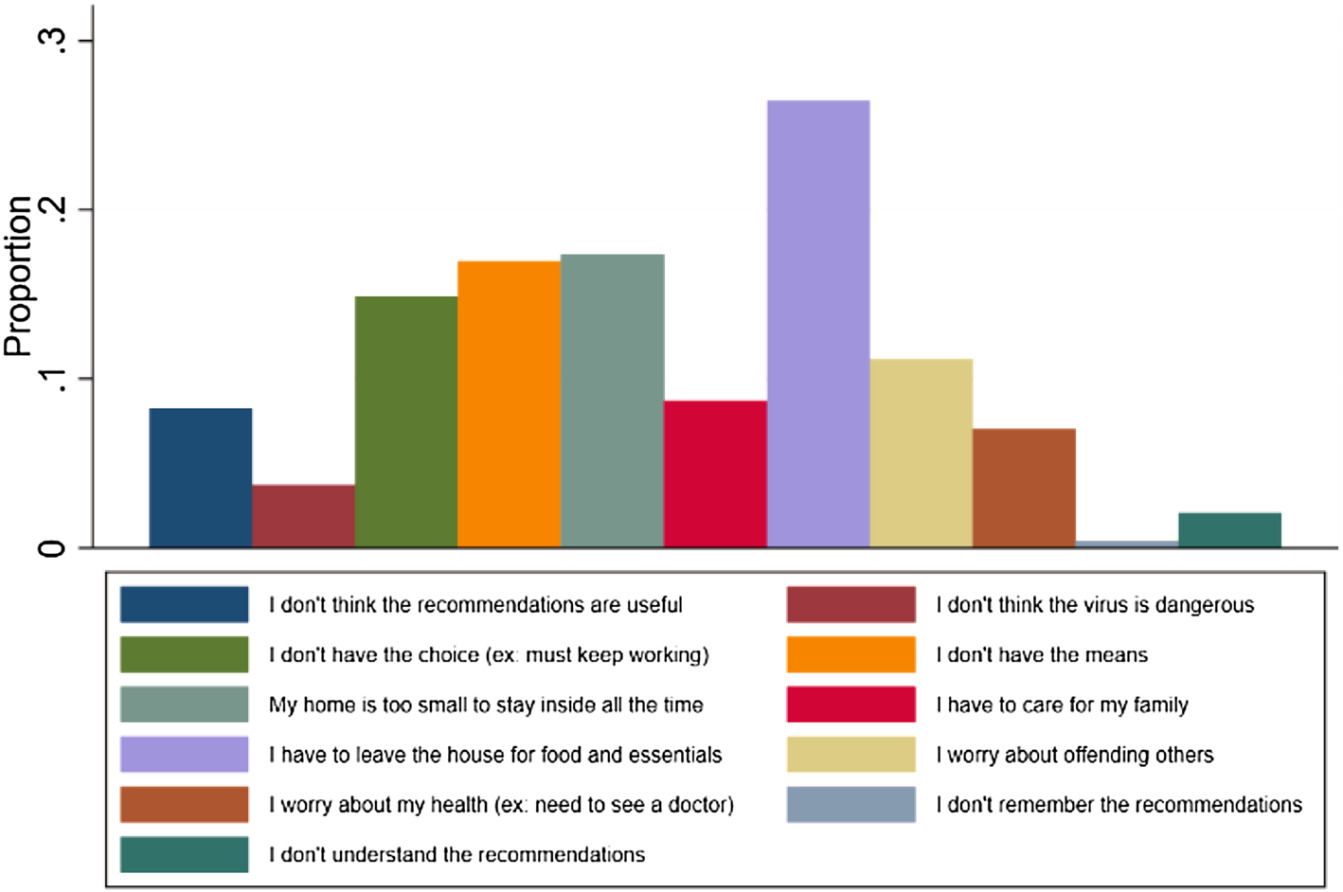
Reasons to stop following COVID-19 recommendations

The main reason for not following health recommendations was “*the need to leave the house for food and essentials”* (26.4%). In a non-adjusted analysis, this reason was statistically associated with a higher socioeconomic position. Indeed, rejected asylum seekers (naOR 0.34, 95% CI 0.15–0.76, p < 0.01), asylum seekers with lower health literacy (naOR 0.36, 95% CI 0.19–0.69, p < 0.01), lower French language proficiency (naOR 0.48, 95% CI 0.25–0.93, p = 0.03) and asylum seekers living in a community center (0.46, 95% CI 0.26–0.83, p = 0.01) were all less likely to stop following COVID-19 measures due to this reason. Another important reason to stop following COVID-19 measures was “*a too small home to stay inside all the time*” (17.4%) (Fig. 4) . No association were found between this reason and the sociodemographic characteristics of participants (See Table 3).

### COVID-19 Rumors

First, 39% of participants agreed with at least one alternative theory (categorized here as COVID-19 rumors without evidence) about the origin of the new coronavirus or the origin of the pandemic (Fig. 2; See Table 2).

After adjustment for age, gender and education, rejected asylum seekers were statistically more likely to think that “*The effects of the coronavirus have been intentionally exaggerated so that governments can better control their populations*” (aOR 2.81, 95% CI 1.24–6.36, p = 0.013).

Similarly, rejected asylum seekers (aOR 0.32, 95% CI 0.14–0.75, p = 0.008) and participants with a lower French language proficiency (aOR 0.31, 95% CI 0.15–0.63, p = 0.001) were less likely to believe that “*The new coronavirus occurred naturally due to mixing of human and animal viruses*”.

## Discussion

In this cross-sectional survey of asylum seekers in Switzerland during the Covid-19 pandemic, almost half of the participants had low knowledge regarding COVID-19 measures, despite high self-reported adherence and satisfaction with the recommendations. The need to leave the house for food and essentials was the main reason for not following health recommendations. In addition, participants identified television, social media and government website as the primary sources of information about COVID-19 health recommendations. Moreover, living in a community center, being a rejected asylum seeker or having lower French language proficiency were significantly associated with lower knowledge and stronger beliefs in COVID-19 rumors.

A similar study conducted among the general population of the same region (Canton of Vaud, Switzerland) showed a high level of knowledge among 67% of participants (versus 43% in our study) [28]. This difference is most likely caused by differences in educational attainment, health literacy level, French language proficiency, and consequently access to and comprehension of information. Our results are also consistent with other surveys assessing COVID-19 knowledge, notably among Afghan and Syrian refugees in Germany [30], Somali, Karen and Latinx community members in the US [31] or Syrian refugee women in Jordan [32] .

Second, although participants described a high adherence to recommendations, almost 40% believed at least one COVID-19 rumor. This result confirms previous data from male migrant workers in Singapore, where authors found a high rate of participants believing in COVID-19 rumors [33] .

Social determinants such as housing conditions (community center vs private apartment), legal status and language barriers (low French language proficiency) were associated with lower knowledge and belief to rumors. These factors should be considered in health decisions related to the COVID-19 pandemic to mitigate health inequities [34, 35] .

Our study also describes the means of communication used by asylum seekers and refugees to access to COVID-19 recommendations. It is interesting to note the critical role of community interpreters for participants living in community centers where access to other means of information such as television is limited.

Our findings suggest that linguistically and culturally adapted communication seems essential to improve asylum seekers' and refugees' knowledge and adherence. Participatory approaches through community engagement and co-production could be helpful to actively build trust and strengthen public health campaigns, such as COVID-19 vaccination [36–38].

Our study has some limitations. First, the observational cross-sectional design of our study precludes temporal or causal interpretation of the observed associations. That being said, the cross-sectional findings provide a basis for further research on equitable pandemic responses. Second, the survey translations were not back-translated or tested for concordance with the original French questionnaire, although proofreading by another translator was possible for most of the languages translated. Third, the self-reported questionnaire may be subject to desirability bias. This bias is, however, limited by the anonymous nature of this survey. Fourth, our study may have potential confounding biases. Even though we adjusted for a range of potential confounders, it is possible that other factors not considered may interfere with the results. Notably, we have not assessed the cultural backgrounds of the participants. Fifth, our study has potential selection bias. Participants may have a higher degree of integration in society than the overall population of asylum seekers in the canton of Vaud. However, through our recruitment method and the translation of the questionnaires into nine languages, we hoped to limit selection bias. The proportion of participants with a low French language proficiency or in a very precarious social situation (rejected asylum seekers) suggests that this bias is likely limited.

In conclusion, the burden of the pandemic on asylum seekers and refugees is partly related to issues of understanding health recommendations, access to information and the consequences of health restrictions on their daily lives. And this access to information about recommendations and the belief to rumors are associated with language barriers, socioeconomic living conditions and legal status. Therefore, better anticipation of asylum seekers' specific communication and information needs in future public health crises is required. More systematic use of community interpreters or the involvement of communities in disseminating public health messages are potential solutions to tackle those issues and limit the spread of misinformation. Similarly, identifying specific social networks used by asylum seekers could facilitate the dissemination of targeted public health messages. However, further studies, including studies in other countries and longitudinal analyzes, are required to understand better the issues of access to COVID-19 information among asylum seekers and refugees.

### New Contribution to the Literature

Our study found that asylum seekers living in community centers or with language barriers were at risk of health inequities related to poor access to or understanding of COVID-19 public health recommendations. Our study underlined the importance of tailoring public health recommendations and interventions to reach vulnerable populations and considering social determinants of health such as living conditions or language barriers in managing the COVID-19 pandemic among asylum seekers. In addition, findings suggested that more systematic use of community interpreters could help spread public health recommendations more efficiently.

## Supplementary Information

Below is the link to the electronic supplementary material.

Supplementary file1 (JPG 100 KB)

Supplementary file2 (JPG 155 KB)

Supplementary file3 (JPG 208 KB)

Supplementary file4 (JPG 199 KB)

Supplementary file5 (JPG 111 KB)

## Data Availability

The datasets used and/or analyzed during the current study are available from the corresponding author on reasonable request.
