# Supplementary figures and images for: Asylum Seekers’ Responses to Government COVID-19 Recommendations: A Cross-sectional Survey in a Swiss Canton
Source: J Immigr Minor Health. 2022 Dec 12;25(3):570–9. doi: 10.1007/s10903-022-01436-3 (PMC9743178; doi:10.1007/s10903-022-01436-3)

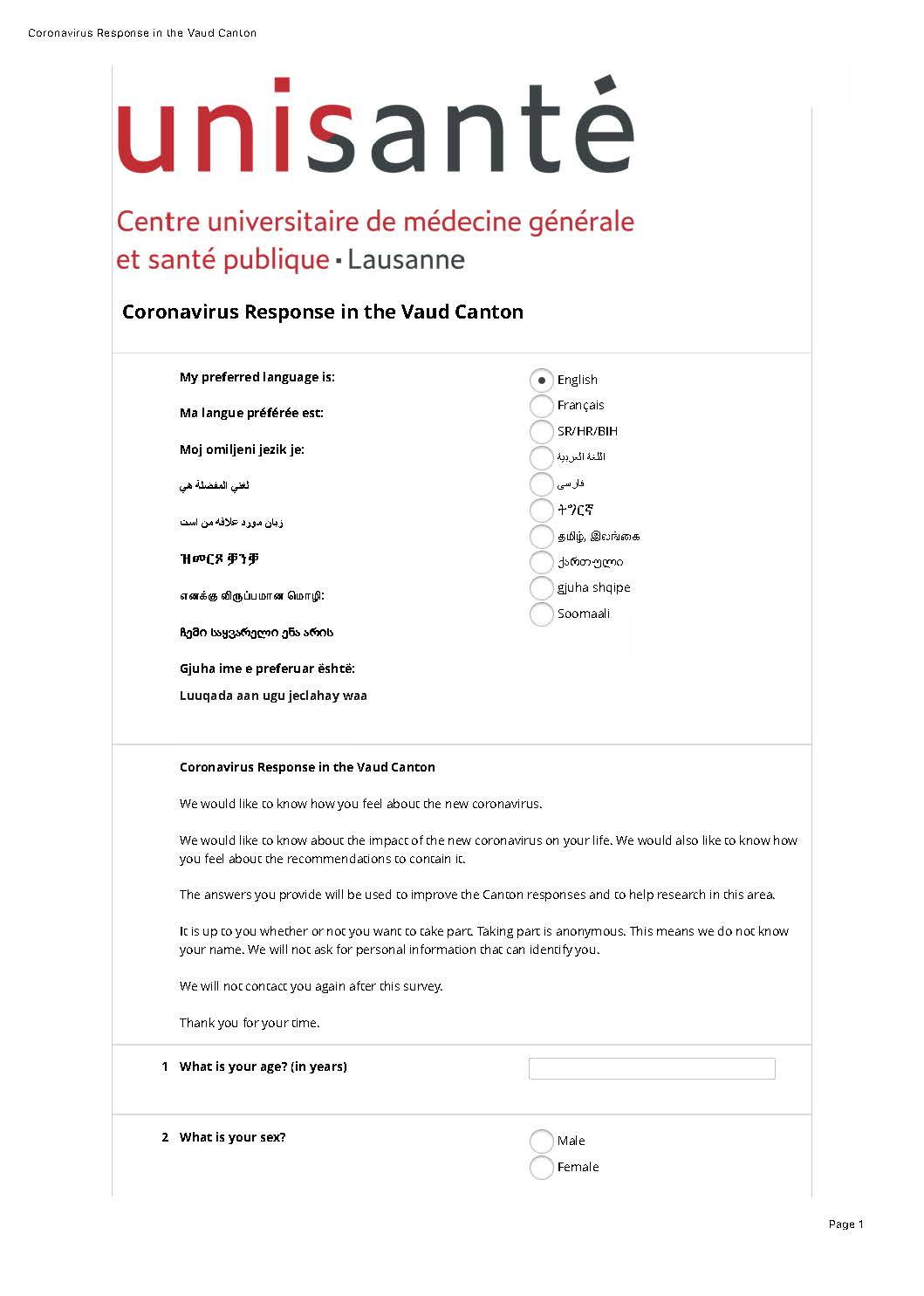

Supplement: Supplementary file 1 — Supplementary file1 (JPG 100 KB) [file 10903_2022_1436_MOESM1_ESM.jpg]

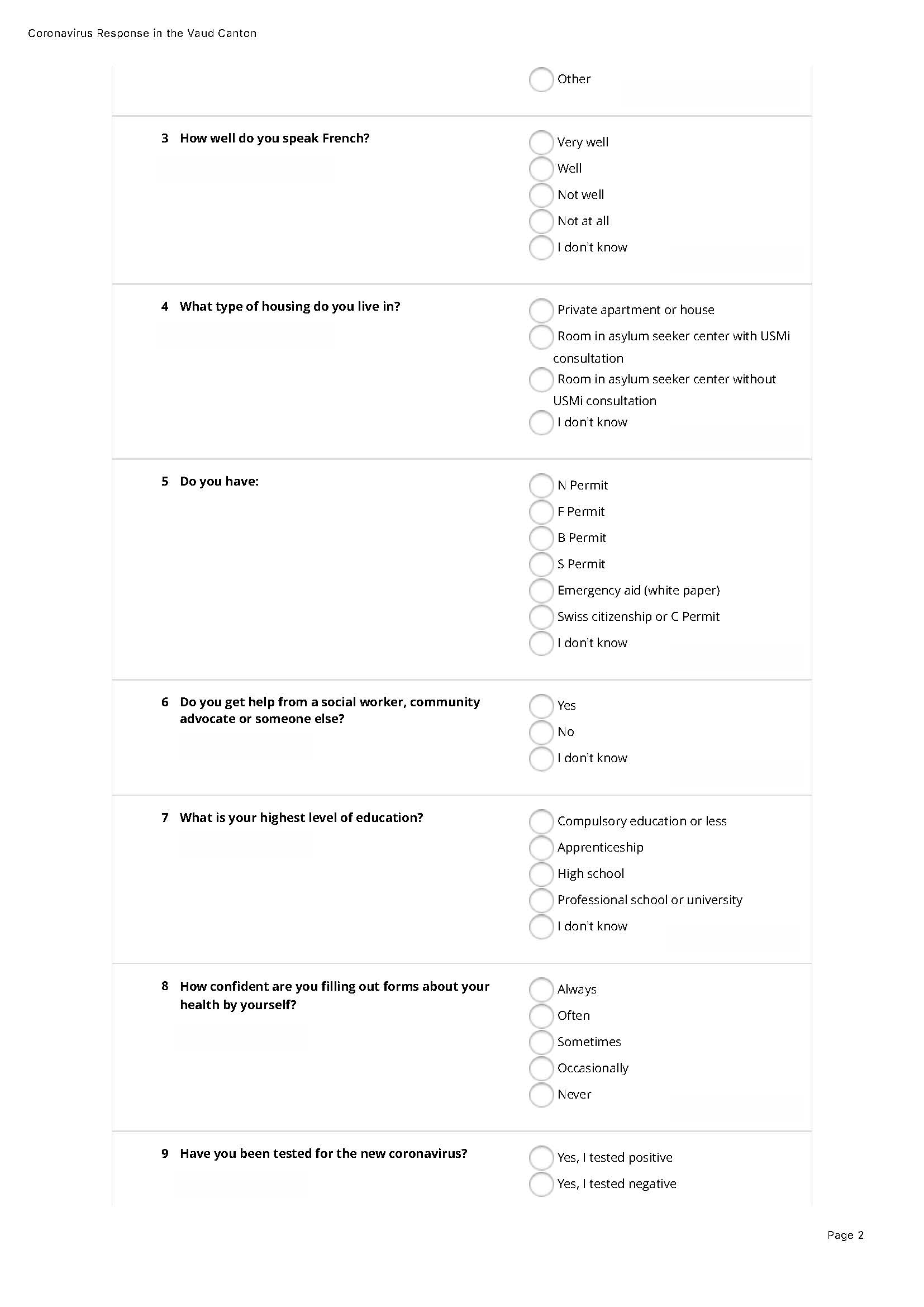

Supplement: Supplementary file 2 — Supplementary file2 (JPG 155 KB) [file 10903_2022_1436_MOESM2_ESM.jpg]

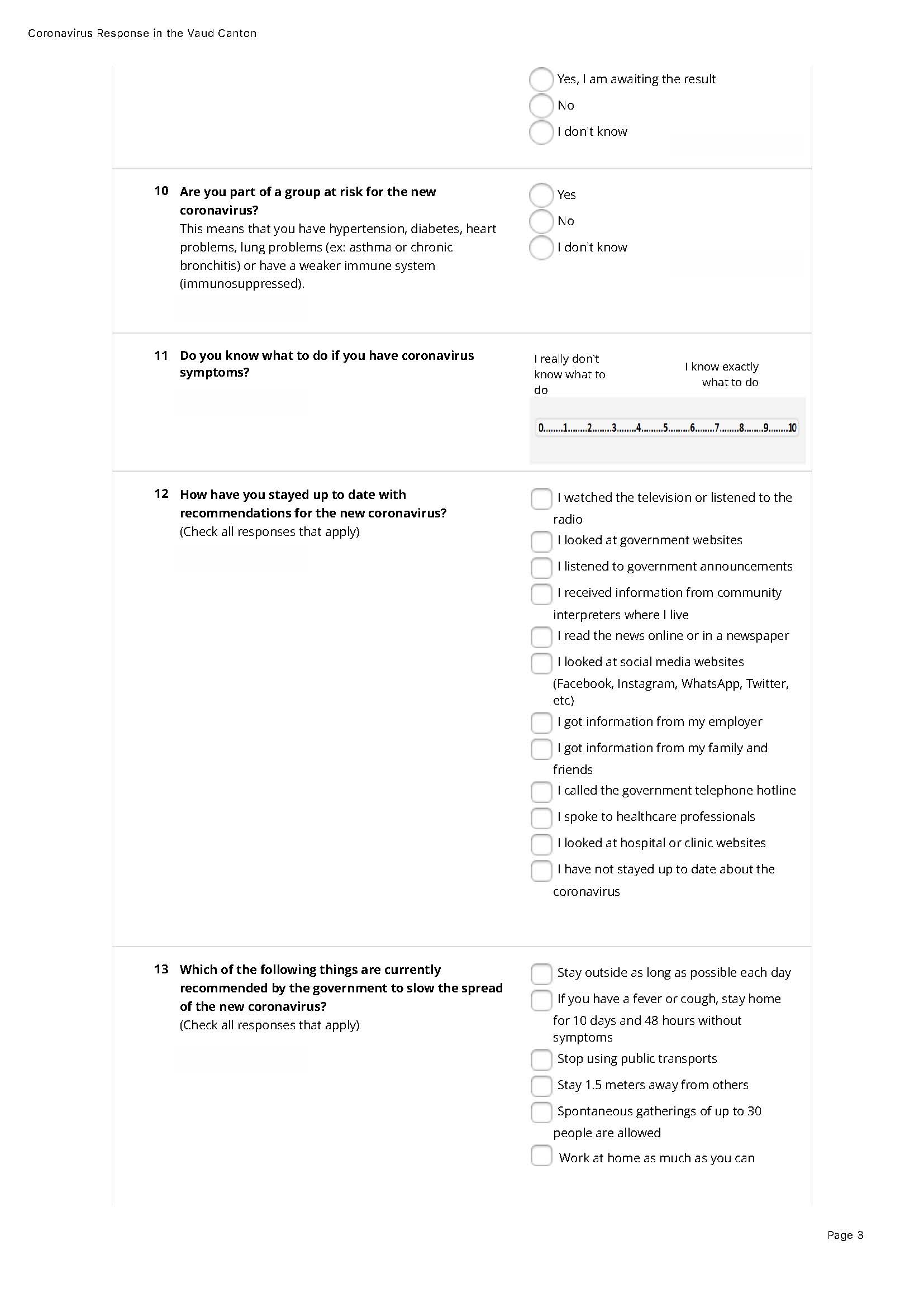

Supplement: Supplementary file 3 — Supplementary file3 (JPG 208 KB) [file 10903_2022_1436_MOESM3_ESM.jpg]

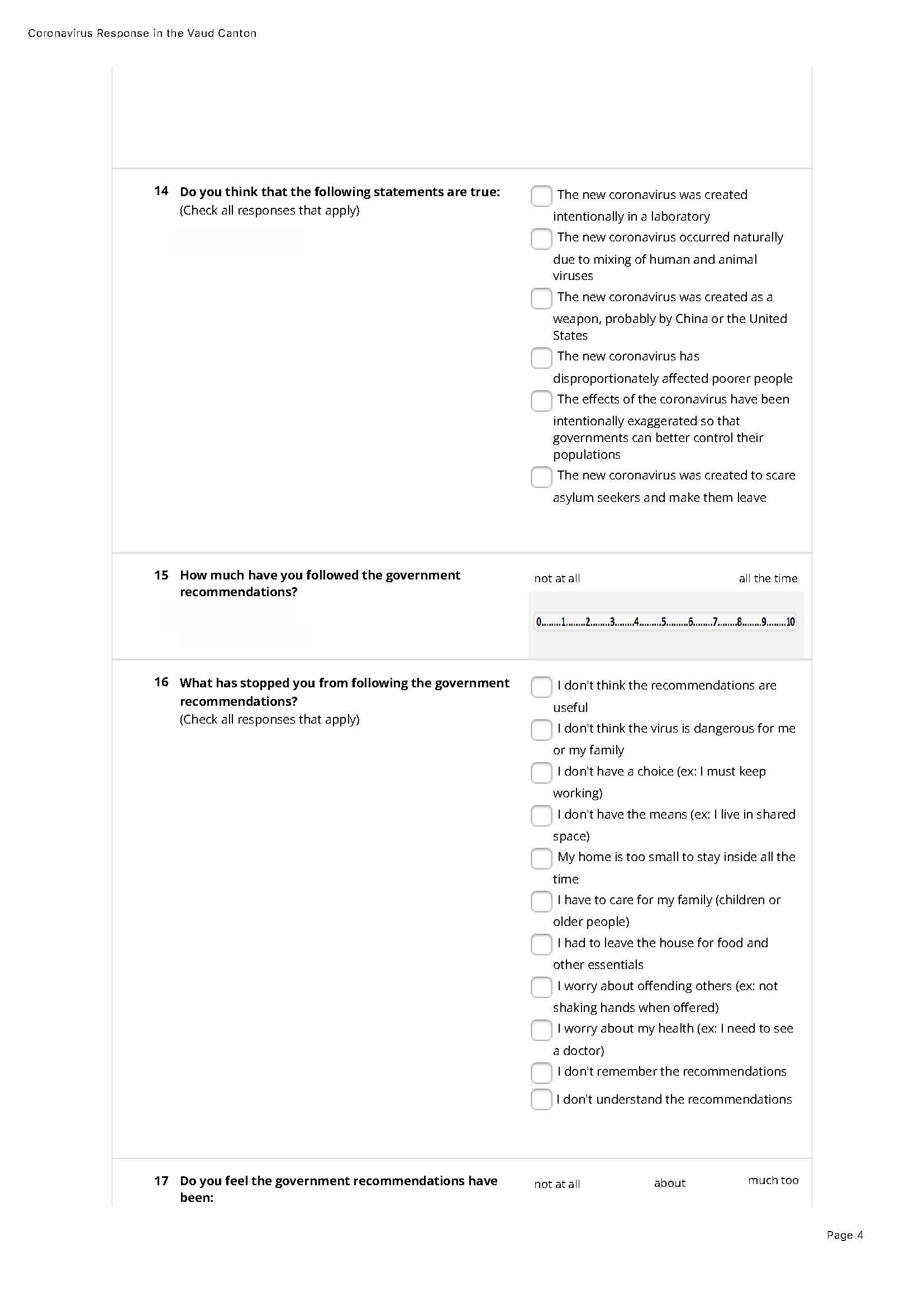

Supplement: Supplementary file 4 — Supplementary file4 (JPG 199 KB) [file 10903_2022_1436_MOESM4_ESM.jpg]

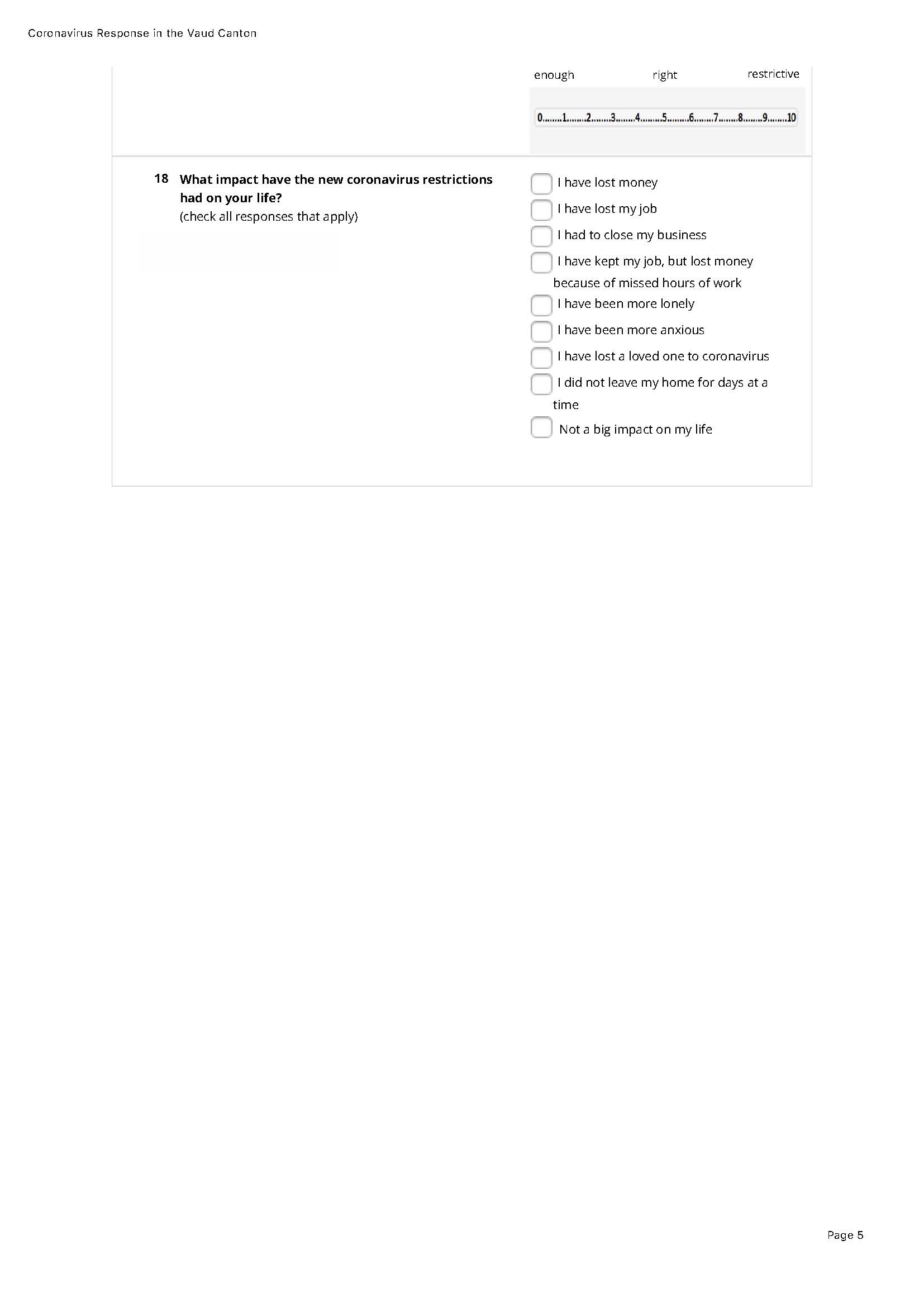

Supplement: Supplementary file 5 — Supplementary file5 (JPG 111 KB) [file 10903_2022_1436_MOESM5_ESM.jpg]
